# A new temporal framework for the passionate engagement journey of ultra-endurance athletes: A qualitative investigation

**DOI:** 10.1371/journal.pone.0293864

**Published:** 2023-11-27

**Authors:** Tatjana Bill, Roberta Antonini Philippe

**Affiliations:** Institut des Sciences du Sport, Faculté des Sciences Sociales et Politiques, Université de Lausanne, Lausanne, Suisse, Switzerland; Universidad Cooperativa de Colombia, COLOMBIA

## Abstract

The explosion of popularity of ultra-endurance (UE) sports in the last 20 years has attracted a lot of interest amongst sport psychologists who studied many aspects of their participants. However, a temporal perspective on the development of the UE engagement was missing. With this research we aimed to explore the long-term individual journeys of UE athletes and how their passion developed over time from the moment of inception to full adoption of UE as an identity and a lifestyle. For this purpose, we conducted semi-structured interviews with 16 UE athletes (12 male and 4 female, mean age 43.4, SD 10) in cycling, trail running and triathlon. Five key themes (with 13 sub-themes) of their UE engagement journey emerged as the result of the interpretative phenomenological analysis of the interviews: 1) The making of the UE athlete, 2) Finding the tribe, 3) Peak experiences, 4) UE lifestyle and 5) UE passion and outcomes. Rich descriptions were generated demonstrating that the development journey of UE athletes is a multi-year, sometimes life-long endeavour, which is full of unique experiences, emotional rollercoasters, passion, overcoming and surrender stories and also of spiritual growth. And while each journey we heard was unique, we could observe similarities in its key milestones. We summarized them in a wholistic UE journey framework which we developed based on this research. The new “Temporal framework for progressive UE engagement and passion development” is presented here for discussion and further validation.

## Introduction

Ultra-endurance sports and races are experiencing an unprecedented popularity growth among non-professional athletes globally [1]. The unique demands of extreme races and the psychological characteristics of UE athletes have attracted a lot of interest from academia. Studies were performed to analyse the personality [2–4], traits [5, 6], implicit motives and basic needs satisfaction [7], need thwarting [8], cognitive functioning [9], key motivations [5, 10], mindfulness [11], mental toughness [12, 13], psychosocial factors as predictors of drop-out [14], social interactions [15], consumption of extraordinary experiences during the race [16], withdrawal stories [17], vitality states [18], pain perception [19], energy deficiency [20], performance predictors [21], sex differences in physiology [22], potential long-term health problems [23], mood variability [24], etc.

The identity research in sports psychology until now primarily focused on athletic identity, which is defined as the “degree to which an individual identifies with the athlete role, within the framework of a multidimensional self-concept” [25]. An athletic identity is developed through the acquisition of skills, confidence, and social interaction during sport and thus plays cognitive and social roles. This concept, and especially its measurement tool, the Athletic Identity Measurement Scale [26] is widely used to evaluate motivation and dedication to sports in professional and elite sports. However, out of 108 empirical studies on athletic identity selected for meta-analysis [27], none was done on the sample of non-professional UE athletes.

Several studies also applied a broader social identity construct to UE sports. Thus, in a qualitative interview-based study with Polish winter ultra-marathoners [28], researchers looked into motives for participation, subcultures and authenticity of the experience. They presented ultra-running as one of the most significant phenomena of our time and of Western societies and suggested that a sports event offers participants an opportunity to create and stabilize their own identity (a value in itself) by means of an extraordinary experience of a collective nature largely missing in individualistic cultures, which is a thesis we fully support. Social identity theory was also used in a large sample study (n = 916) with UE gravel cyclists [29] which found that social identity had a significant direct relationship with motivations, constraints and negotiation strategies and a significant indirect effect on planned participation. Both studies highlight the importance of the social identity construct to better understand the psychology of UE athletes and the need to apply broader theories and frameworks to study the complex factors leading to the committed engagement with extreme and UE sports. A recent study on UE runners used the framework of reversal theory to understand why and how humans run ultra-marathons [30], generating interesting phenomenological insights into meta-motivational states and the psycho-diversity of UE runners.

And still, in spite of a lot of research light shed on the selected topics of UE, a general psychologic theory or model explaining why and how humans conquer ultra-distances is missing, as well as a long-term perspective on the total journey. The latter is especially relevant in UE sports, where the engagement of non-professional athletes can last a lifetime. The hobby becomes a passion and a lifestyle and seems to dominate the self-identity definition and to take the shape of personal ideology [31]. Which theory of human behaviour can explain the journey of UE athletes and which assumptions about human nature should build the theoretical foundation for deep qualitative research of the UE phenomenon? And how can passion and identity development be investigated on a time axis while balancing the narrow view of individual lived experiences with the broad philosophical perspective on the UE phenomenon in post-industrial society?

In order to answer this challenge, we merged the psychological and psycho-social approaches of personality theories to gain a broader perspective on the process of the integration of UE passion into the personal identity and lives of non-professional UE athletes. The Self-Determination Theory of Behaviour (SDT) [32] was identified as a meta-theory for the theoretic foundation of this research. It fulfils the humanistic-existential and developmental criteria and enables the psychological, personal and psycho-social perspectives. Being grounded in humanistic and phenomenological psychology and existential philosophy, this theory provides the highest level of theoretic underpinning for this research [33–35]. Its starting point is “the postulate that humans are active, growth-oriented organisms who are naturally inclined toward integration of their psychic elements into a unified sense of self and integration of themselves into larger social structures” [32].

According to this theory, humans are driven by the innate psychological needs for autonomy (a desire to feel a sense of personal initiative), competency (a desire to interact effectively with the environment) and relatedness (a desire to feel connected to significant others). The fulfilment of these needs is seen as the necessary condition for ongoing human growth, integrity and overall well-being and all three of them must be satisfied for optimal human development. So “people will tend to pursue goals, domains, and relationships that allow or support their need satisfaction” [32].

Naturally, people are rarely consciously aware of their psychological needs and they don’t pursue their needs satisfaction per se. They are rather after activities and goals which appear interesting, important and appealing to them. Therefore, we assume that a UE race represents such an important activity, and the performance goal in a race represents a “growth goal” [36] which enables athletes to fulfil their needs: experience the autonomy, expand their competence, and enjoy the relatedness, camaraderie and care within the UE community.

We also assume that engagement in such an intense hobby as UE training and racing involves a strong motivation and a passion. The Dualistic Model of Passion by Vallerand [37], which follows the SDT, assumes that people engage in various activities throughout life in the hope of satisfying the basic psychological needs of autonomy, competence, and relatedness–and some of these activities become their passion. “Passion can be seen as a strong inclination toward a specific object, activity, concept or person that one loves (or at least strongly likes), highly values, invests time and energy in on a regular basis, and that is part of one´s identity” [37]. The more the activity is valued and the more it is important, the more it is likely to become a part of one´s identity [37].

There are two types of passion according to the Dualistic Model of Passion: Harmonious Passion and Obsessive Passion [38]. Whereas both passions are deeply integrated into the core concept of identity, they differ by how they are internalized. Harmonious passion results from an autonomous internalization of the activity in one´s identity and is a free and flexible choice, and is therefore internally motivated. Obsessive passion refers to a controlled and external internalization, which creates negative affect and an internal pressure to engage in the activity, without considering conflicts it creates. Harmonious passion can fuel motivation, create flow, increase well-being and provide meaning in life, whereas obsessive passion typically interferes with a balanced life by creating negative emotions and conflicts with other aspects of life [39].

Obsessive passion may lead to rigid persistence in a physical activity, leading to chronic injuries [40] and also to dependence on sport and exercise [39, 41]. Harmonious passion was also related to exercise dependence, but to a lower degree [41]. Obsessive passion was found to be associated with lower quality of relationships with teammates [42, 43] and negative impact on team cohesion [44]. Interestingly, one condition was found to lead to higher levels of well-being for an obsessive passion than for a harmonious one: it is the situation of person-environment fit [45]. It occurs when characteristics of the individual and those of the environment match. Obsessively passionate athletes thrive in highly competitive environments, such as training and selection camps, while harmoniously passionate individuals prefer less competitive and demanding ones [45].

Both social and personal factors contribute to the development of passion. Before they find something interesting and relevant, most people engage in a variety of activities (especially when young). The process goes from activity selection, to activity valuation to activity internalization [32], which supports the idea that UE engagement happens on a time continuum and is shaped by personal and social factors in the process.

Nowadays, and increasingly over the past 20 years, UE has become a passionate and a high value activity for millions of people. It is also a part of identity for millions of people. They all went through a certain personal journey and very unique experiences leading them to develop this passion and adopt a certain lifestyle, which differs significantly from the norm. The aim of this study is to explore the whole UE journey of individual athletes from the moment of initial interest to a fully developed passion and to better understand the factors impacting the formation of UE passion and adoption of UE as a personal identity and a lifestyle. The novel aspect is the long-term view on the UE phenomenon in a life journey format, as seen from the perspective of lived experiences of UE athletes, as well as an attempt to create a generalized holistic framework for this journey as well as a model explaining the long-term UE engagement and passion development.

## Materials and methods

### Participants

For this study, 16 non-professional UE athletes in trail running, triathlon and cycling were interviewed. They were selected based on the following criteria: 1) participation in UE races (minimum of 2 races lasting over 6 hours over the past 3 years) as an amateur (i.e. not professional) athlete, 2) interest in the topic and availability for interview, 3) English speaking. While this purposeful sampling was to serve the explorative and not representative needs, a variety of ages, years of experience and a balance of genders was aimed at.

The participants were an average 43 years old (SD = 10), 75% male and 94% highly educated (bachelor degree or higher). They practiced UE as their main hobby for an average of 14 years (SD = 8.7), all worked in white-collar knowledge-based professions, almost all full-time (only two on 80% basis)–see Table 1. Nine participants (56%) were married or in a relationship, seven were single. Four participants had children living with them.

**Table 1:** Participants´ demographics.

	Age	Gender	Nationality	Education	Sport	Years in Sport
**A1**	46	M	TUR	Master	Triathlon, trail running	11
**A2**	45	M	ITA	Master	Triathlon	17
**A3**	61	M	USA	Bachelor	Triathlon	35
**A4**	43	F	CAN	Master	Trail running, cycling	20
**A5**	41	M	CHE	Master	Trail running	10
**A6**	24	M	CHE	Master	Triathlon	4
**A7**	37	M	AUS	Master	Trail running, triathlon	7
**A8**	51	F	CHE	Vocational	Triathlon	26
**A9**	48	M	DEU	PhD	Triathlon	10
**A10**	62	M	GBR	Bachelor	Triathlon	20
**A11**	36	M	GBR	Master	Trail running	7
**A12**	33	F	AUT	Master	Trail Running	20
**A13**	37	F	NLD	PhD	Trail Running	8
**A14**	46	M	ESP	Master	Trail running	18
**A15**	35	F	AUT	PhD	Triathlon	4
**A16**	50	M	GBR	Master	Triathlon, cycling	6

### Procedure

This project obtained ethical approval from *La Commission cantonale d’éthique de la recherche sur l’être humain (CER-VD)* of the canton Vaud, Switzerland, prior to starting the study. Participants were recruited via the personal network of the principal investigator, were recommended or responded to advertising on social media and on a sports media platform. In the recruitment communication, the aims of the research were outlined and interested parties were encouraged to make contact if they wished to volunteer for the study. Those who responded to the recruitment appeal received an Information Sheet for Research Participants, with interview information and instructions in Q&A format. They also received and signed an Informed Consent Form before they were interviewed. Participants were reassured that (a) participation in the study was strictly voluntary, and (b) the collected data would only be used for research and would remain strictly confidential. The transcripts got anonymized and coded under the following identifications: A1, A2, A3, etc. The voice records were destroyed after transcribing and the transcript data was stored on an academic server based in Switzerland in accordance with the Swiss Federal act on data protection (FADP 235.1) and the Canton of Vaud Act on data protection (LPrD 172.65).

### Research design

The theoretical foundation of the conducted research lies in the constructivist paradigm and phenomenological philosophy. Together they define the methods of the presented research. Qualitative interpretative phenomenology was selected as the methodology of choice for our research question due to its focus on understanding and interpreting the lived experiences of individuals. Being an epistemologically consistent one [33, 34], it informed our sampling, data collection and data-analysis choices. Thus, we employed a purposeful sample of non-professional UE athletes with maximal variation of key demographics (gender and age) to get a variety of experiences, used semi-structured interviews to collect the data and tools of interpretative phenomenological analysis (IPA) and hermeneutic reduction to analyze the interview data qualitatively.

### Interview

Each interview followed a semi-structured guide with 30 questions (25 open and 5 closed), lasted between 1.5 and 2 hours and took place face-to-face, respecting Covid-19 protocols, or via Zoom. The interviews were designed to encourage story-telling around key moments of the UE journey, to stimulate self-reflection, elicit emotions, and evaluations of key experiences. As a visual help and a structuring tool for temporal storytelling, participants were asked to draw a timeline of their EU journey and its key events, starting from its inception point, freely chosen, to the current time and beyond–by projecting it to the future in line with their vision of future UE engagement–and to talk about it in as much detail as possible. Respondents found the exercise interesting and novel, stating that it was a learning opportunity for them. Please see S1 and S2 Figs in Supporting Information with 2 examples of timelines created by participants.

When talking about self-identity and UE lifestyle, the researcher used the stimuli of 16 photo collages, with images of different UE athletes and also humans in different social roles (parenting, working, etc.) and leisure situations (travelling, relaxing,) as well as objects (food, fashion, etc.) to facilitate the discussion. Please see S3 Fig in the Supporting Information for the photo collages used. Participants were asked to select several collages which best represented their self-image and to explain their choices. All participants liked this exercise and it helped to create better rapport between researcher and participant, contributing to a joint construction of interpretations and thereby the ontological authenticity of the research [46, 47]. Per se, right “chemistry” between the participant and the researcher is a key factor for the quality and success of the qualitative research. It facilitates the creation of new understanding and knowledge by constructing new interpretations of lived experiences and by generating “rich descriptions” of the studied phenomenon [48].

### Interview data processing

Interview data was recorded and transcribed verbatim with total interview transcription data comprising 139 pages and 77104 words. The analysis of the interview transcripts was done using the IPA and iterative hermeneutic reduction techniques, whereby the athletes´ accounts were analyzed and interpreted by the researcher, in line with the “double hermeneutics” [48] process inherent to this type of research.

The step-by-step analysis of interviews followed the 6 phases of thematic analysis proposed by Braun et all, [49].

**Fig 1:**
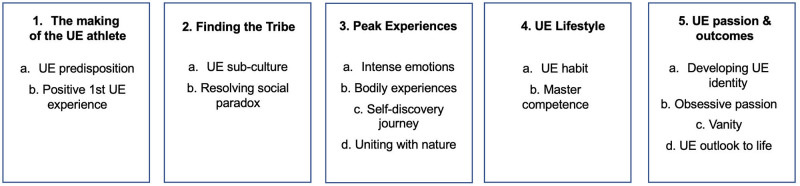
Themes and sub-themes from interviews with non-professional athletes.

Phases 1–2: Familiarization & coding—Deep engagement with data consisted of multiple readings of the transcriptions, using the analytical and meaning-making lens of the researcher. A wide set of creative codes and notes was used to tag the transcripts, preparing the foundation for the theme development.
Phases 3–5: Theme development, refinement and naming—These phases consisted of 2 rounds of analysis of the pre-coded interview parts. The 1^st^ round of analysis focused on identifying rich descriptions of key events, looking for connections and shared themes and analyzing them using the phenomenological reduction technique of empathic yet critical engagement [50]. A special focus was put on the narrative of UE stories shared by participants and the analysis of identity positioning through it [51]. In the 2^nd^ round the key subthemes with verbatim statements describing them were extracted, yielding a total of 85 sub-themes. With this list in mind and at hand, the transcriptions were read and analyzed one more time, looking for patterns, connections and tensions. As a result, the 85 sub-themes were organized into 5 themes and 13 sub-themes, which all received the descriptive names presented in Fig 1.
Phase 6: Writing up—At the final stage of the IPA analysis, the analytic themes were transferred into a narrative account, enriched by the verbatim statements, which is presented below in Results.

**Fig 2:**
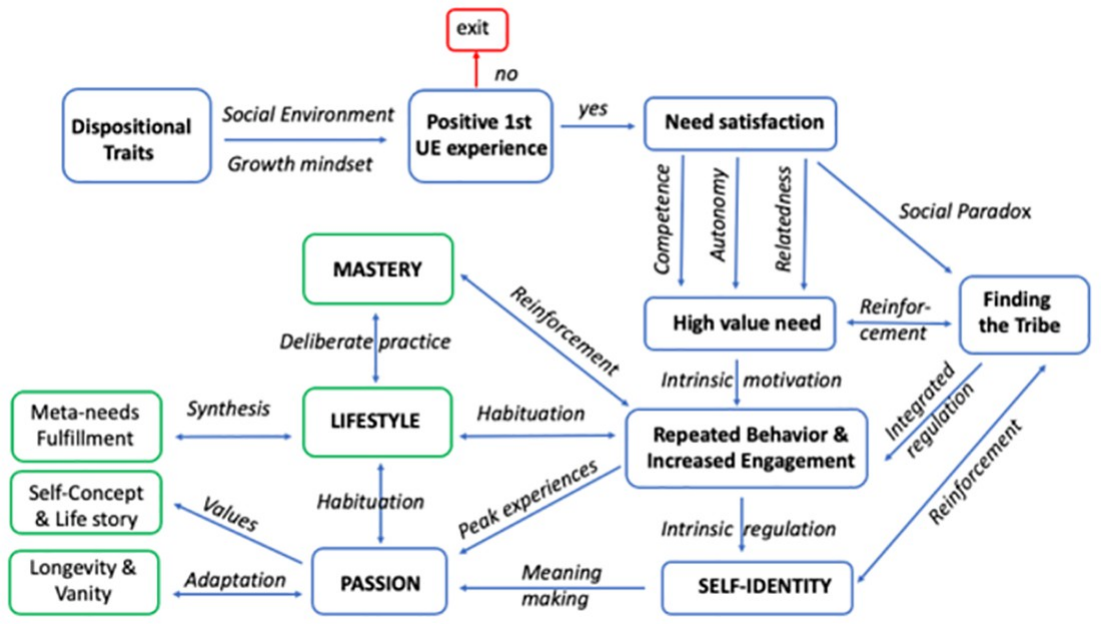
A temporal framework for progressive UE engagement and passion development.

With 16 interviews done, we clearly reached “data saturation” [52] and could observe repeating patterns and milestones. Therefore, we added an additional ideation step to our analysis in order to conceptualize the data and try to come up with a theoretical framework for the UE journey and a model describing and possibly explaining it. We used the Thematic network for psycho-diversity of running experiences and motivations [30, p.5], Graphical summary of structural model results [28, p. 95], Social identity of ultramarathon runners scheme [28, p. 3] as well as the comprehensive integrative “The New Big 5” framework of personality [53, p. 10] as examples. Specifically, we merged the Dualistic Model of Passion and Self-Determination Theory of Behaviour with qualitative data presented in Results, as well as with the studied literature and knowledge of researchers to create “The new temporal framework for progressive UE engagement and passion development” which we present in Results, Fig 2.

### Role and rigor practice of the researcher

Inherent to interpretative phenomenology is the concept of the inseparableness of the researcher and the process and results of inquiry. The existing knowledge and opinions of the research needs to be made explicit, understood and interpreted in the course of analysis. Thus, the researcher practices “double hermeneutics”, making meaning of participant experiences and own experiences. With the principal researcher being a former UE athlete, three main procedures have been implemented to ensure the validity and rigor of qualitative research [46]. First, a self-interview was done and recorded before the analysis phase, to make the personal UE journey explicit. Second, reflexivity was practiced during each stage of the analysis on social and cultural factors and forces, as well as personal experiences, belief system and background, which can influence interpretation. Third, transcripts and draft article were shared with 4 interview participants: member-checking for accuracy of transcriptions and member reflections for enrichment of interpretations. No issues were identified in all three procedures while feedback from reflections was contextually integrated into the manuscript.

## Results

The following themes and sub-themes were identified as a result of the IPA analysis of interviews with 16 non-professional UE athletes:

### Theme 1: The making of the UE athlete

In the beginning of each interview and after the warm-up discussion about the current and past seasons, each participant was asked to share the origin story and to recall their first encounter with sports in general and their first experience with UE racing specifically. While each story was unique, two common sub-themes crystallized and repeated themselves in each interview: first, participants identified themselves as someone with a UE character, or predisposition tracing back to early childhood, and second, they all had a positive 1^st^ experience with UE racing.

#### UE predisposition

The majority of participants (14 out of 16) described themselves as “sporty kids”, growing up playing sports and spending a lot of time outdoors. Several had adults in their childhood, who were either sporty or outdoorsy themselves and who influenced and/or supported them in their early sporty endeavours. This urge to move persisted later in life, transformed into different sport interests, sometimes went dormant for several years occupied with studies, early careers or/and building families, but ultimately resulted in their 1^st^ participation in an UE race. All participants spoke of their “natural” inclination towards endurance sports and how they felt attracted to outdoors, adventure and exploration of own limits. We could also observe several commonalities in terms of psychological traits, such as the ability to practice delayed gratification, engage in deliberate practice, and commit and follow through to reach long-term goals. They all displayed the need for a lot of sensory stimulation as well as a growth mindset. These personality characteristics, the seemingly inborn predisposition to endurance, adventure and outdoor, coupled with a set of circumstances and the presence of crucial friends or groups led them to their 1^st^ UE race.

*“The thing that has always been there is this drive to do extreme sports*.*”*

*(A10)*

*“My dad used to take me on the mountain hikes since I was a child*.*”*

*(A11)*

*"I have a really bad urge to move all the time*, *which I have since my childhood*. *For me*, *it is a torture to sit all day*.*”*

*(A15)*

*“My PE teacher was Tasmania´s best marathoner at the time*. *He saw that I could run*, *he said*, *come down*, *train with us*. *We were only about 12 people*, *kids*, *but only hand-picked ones*. *When I met him*, *he was 60–65 and he coached me until I was 17*.*”*

*(A7)*

*“As a child*, *my parents threw me into loads in sports*. *I played tennis*, *badminton*, *cricket*, *rugby and football at a regional level*. *So I was super sporty and then then went to a boarding school where it is sports all days*, *morning*, *after lunch*, *after homework*. *From 14 to 18 y*.*o*. *At University I played rugby and then I started work and no sport for 4 years maybe*, *or even longer*. *The sport I do now in one month would be double what I did in year*. *And*, *around 2011 was when someone said that I should get into road biking*. *So*, *I bought a road bike*. *The 1st event I did was 100miles ride London*.*”*

*(A16)*

#### Positive first UE experience

All participants reported having had positive emotions and overall, a very positive experience of their first UE race. In fact, for many, the first race represented an overwhelming event, which they all talked at length about, describing the kaleidoscope of emotions and physical sensations which they had never experienced before: from extreme fatigue, physical pain, dealing with elements, thousands of meters of elevation to the über-joy of deep connection with nature and the pure elation of crossing the finish line. Combined, these experiences brought about the re-definition of their perceived limits of physical and mental abilities and represented a huge source of pride and joy and a boost to self-confidence–hence, a very rewarding experience overall.

*“Oh*, *it was great (my 1st Ironman race)*. *It was actually amazing because I did not know what to expect and I really enjoyed it*. *I don’t say it was easy*, *I suffered of course*, *but it just went like this*. *It was an amazing experience*.*”*

*(A8)*

*“I felt this jubilation crossing the finish line*. *When you feel that*, *tears well up and you feel*, *well*, *that I have achieved something special*, *only I can do it*, *I have done it*, *I did not know my body could do this*, *I did not know my mind could do it*.*”*

*(A7)*

*"The best one is really the 1st race*, *the Ultracks in Zermatt*. *When you realize that you can do that and you feel good*, *and you suffer a bit*, *but you suffer in a healthy way*. *And I thought that I was not sure that I could do such a race because if you never went and did an 8hr race*, *you don’t know if you can do it*. *It is 3600m of elevation*, *it took me around 7hr 40min*. *And it was a really cool moment when I realized that I can do it*. *It was cool*, *it was possible*.*”*

*(A15)*

Results show that “The making of the UE athlete” requires “the right material” of a person with an innate predisposition to UE and adventures to be exposed to the UE activity and who attaches a positive meaning to the 1^st^ UE experience, in spite of difficulties faced. This 1^st^ UE experience represents the first crucial milestone on the journey to long-term UE engagement.

### Theme 2: Finding the tribe

The social component plays a crucial role in the stories of UE athletes. On the one hand, they experience a lot of pressure for not conforming to traditional roles in society, especially felt by women and older athletes, whose lifestyles and pastime differ significantly from their peers. On the other hand, they are looking for their “tribe”, for people who are like them, to get social confirmation and acceptance. This contradiction represents a social paradox inherent to UE.

#### UE sub-culture

An idea of being different, an outlier, runs like a red line through all interviews: being a non-professional UE athlete is widely associated with being “crazy” in broad public. The athletes describe themselves as “not normal” or “outliers” and their lifestyles as very different to their peer groups. They talk about an UE sub-culture, a tribe, referring to “us”, i.e. people who train for and race UE events, juxtaposed to “them”, meaning people with traditional hobbies and lifestyles. “Them” are frequently also their own families, who mostly support but actually don’t understand. This creates a lot of pressure, especially on women:

*“Nobody understands it apart from my sports friends*. *Outside people don’t understand it*.*”*

*(A3)*

*“When they hear the distance*, *the things I have done one after another*. *I scare a few people*, *especially men*, *that´s the thing*. *I don’t think I scare off women actually*, *but men I do scare off*. *When I started doing longer distances and I was not getting married and having kids*, *my mom said “why are you wasting your time with the stupid sport*?*” And that was the argument every week “why are you wasting your time*?*” And then I just stopped telling her*. *So*, *I have never told her that I did an ultra-trail race*. *It is a bit weird*. *My parents had other expectations*, *and running around the mountain was not part of their expectations*.*”*

*(A4*).

*“For my mom it was hard to wrap her head around it for a while*. *She never understood why a middle-age guy would be in lycra for almost all of his life*. *But when I started doing races in Turkey and she saw me doing it–she kind of liked it*. *She came to half-Ironman I was doing in Turkey and there was this 70 or 80 years old Russian guy doing it and she was so impressed by it*. *She more accepted it rather than understood it*.*”*

*(A1)*

#### Resolving social paradox

Judgement and lack of understanding in general in the outside world, is, however, more than compensated by the unity within the UE sub-culture. All participants talked about meeting like-minded people in groups or clubs of UE athletes, who were more advanced in UE experience and who inspired them and helped to navigate many decisions at the inception and early stage of their UE hobby. These groups and people serve as a source of information and provide social reinforcement for the “deviant” UE behaviour and lifestyle.

*“When I found Ironman triathletes*, *I finally found my people*. *The mentality of people who enjoy swimming 5km*, *10km*, *biking 200km*. *These are the people that I finally felt and I was at home with*. *That was a huge gift to me*. *My life finally developed reason and calmness*. *And my self-identity*.*”*

*(A3)*

*“My friends told me about Ironman–after the swimming we went for a beer and talked about Ironman*.*”*

*(A2)*

We believe that “finding the tribe” of like-minded people who share the same interests, values and norms and being accepted there resolves the social paradox of the intense UE hobby and represents the 2^nd^ important stage on the long-term endurance journey.

### Theme 3: Peak experiences

Racing a UE race is an extreme event. physically, mentally and emotionally. It is so extreme and intense that it is hard to verbalize. It is truly an ineffable experience. One of the participants tried to describe it:

*“Maybe you can describe things that are less important in your life*. *But when it is very important and very special*, *then you cannot find words*. *It is like when somebody asks me*: *“What did you feel when you saw your daughter for the 1*^*st*^ *time*?*”*

*(A14)*

#### Intense emotions

All athletes reported having experienced intense or peak emotions during UE races. In fact, the tougher the race, the more memorable and extreme emotions it produced and the more fulfilling it was. One of the athletes verbalized it succinctly:

*“I need to suffer to get more back from the race*.*”*

*(A7)*

All participants mentioned the unpredictability of every race, its emotional dualism and polarity (*“It was horrible but nice”*, *A5)* and the endless rollercoaster of positive-negative emotions. Intense negative affect becomes a positive one within a very short time, and then it goes back to negative on the next ascent and the whole chain of emotions repeats. This struggle between body and mind happens in every UE race.

*“But what is really surprising*, *the fatigue and how you feel is really not linear*. *You can feel like shit after 40km*, *but it does not mean that you won’t feel much better 2 hours later*. *I felt this already in other races that you hit some lows*, *but then you eat something*, *you get on top of the mountain and suddenly you feel much better*. *And knowing yourself*, *knowing that you can feel really terrible now and maybe you will feel much better in two hours helps a lot*. *Because*, *psychologically*, *these long races are all about mental*, *much more than physical*.*”*

*(A5)*

In most cases, the extreme hardship of the race is overcompensated by the elation of finishing and the joy of having mastered, persevered and overcome the difficulties. This is also why after finishing in pain and tears and swearing “to never do it again”, athletes find themselves signing up for the next year´s edition literally the next day.

*“I was super happy*, *super proud to finish*, *impossible to describe the feeling*, *and it keeps with you for a few days*, *you are happy for 2–3 days*. *It is like a high*.*”*

*(A5)*

*“During the race (100km 6000m of elevation*, *CCC) was super sick again*, *got to the finish line and cried*. *I sat on the bench and cried*, *asking really what I am doing there*. *And the next day I looked at UTMB (170km race with 10*.*000m of elevation)*.*”*

*(A4)*

#### Bodily experiences

Racing ultra is an extra-ordinary embodied experience. The most profound and mostly negative emotions are triggered by intense bodily experiences, such as extreme fatigue, muscle soreness, blistering, dehydration, indigestion and stomach aches and sometimes light injuries due to falls on technical terrain. One of the interview participants described how he was recovering after the 40 hours, 165km and 10680m of elevation of the SwissPeaks Valais trail running race:

*“It was much*, *much harder than what I anticipated*. *It is a very technical race*, *it has quite steep climbs*. *After the race you almost feel desiccated*. *Even if you drink*, *drink*, *drink the day after*, *you still feel like a dried raisin*. *Completely desiccated*. *I remember my legs—it took me almost a month to stop aching*. *My legs*, *tendons and the joints*… *my knee was swollen*. *Things I have never had before*. *And I had tendonitis in the arms*, *in the elbow*, *from the poles*. *My calf*, *it was like wood*, *for 2 or 3 weeks*. *It took me 2 months to get all the symptoms away*.*”*

*(A5)*

#### Self-discovery journey

A UE race can be a life-changing experience which opens doors to a new level of consciousness and a new and different concept of self. It is the ancient path to enlightenment through bodily suffering, a “beautiful suffering” as athletes call it. For some, it is outright cathartic.

*“I think I learned more about myself in that race than probably in any other moment of my life*.*”*

*(A16)*

*“This was when I discovered something about myself that I knew was there but I was not consciously aware that all my reasons (for racing Ironman) were deeply internal and I had hundreds of them*. *All life experience that I had*, *all the suffering that I had seen*, *all the joy—it all lives there*.*”*

*(A3)*

By pushing the limits of endurance athletes re-define their own physical and mental limits and gain a new level of self-confidence:

“*And after that I knew I could do anything*.*”*

*(A1)*

*“You need to show that you are strong not when things are going well*. *My best race was easy*. *But when things go bad*, *this is where mindset kicks it*. *In general*, *it gave me a mindset to face every obstacle*. *Every challenge that comes my way*. *Growth mindset*.*”*

*(A2)*

An important step on the self-discovery journey seems to be the realization that experiences made in UE training and racing apply to other areas of life. All interviewed athletes reported on a strong connection between UE and other areas of life and on how their self-perception changed under the influence of UE.

*“It helped me to better handle my job*, *to have less stress*, *it gave me energy*. *It gave a way of escaping from some of the pressures of the job*, *so it helped me to do other things*.*”*

*(A10*).

*“I think it changed my life in a positive way*. *No single event has done that*. *This whole journey has been life-changing*. *I am fitter*, *healthier*, *I think I am a better person*, *I am more relaxed*, *I know more about myself*.*”*

*(A16)*

#### Uniting with nature

The bodily experiences are intensified by being in nature. Athletes report on magical moments in the wilderness, on encountering nature´s beauty and experiencing it in a new way. They start appreciating nature more and want to spend a lot of time outside and discover new places as part of their UE hobby:

*“It´s really magical to be going through the night on the mountains almost completely on your own*. *You see the animals*, *it´s pitch dark–and I found it completely magical”*

*(A5)*

*“What I really like is discovering new places in the mountains*. *The biggest reason to do it is to be in all those nice places*.*”*

*(A4)*

The extreme experience stage of the UE journey is probably never completed. It is the essence of the UE phenomenon. However, we believe that fully embracing this experience, attaching deep meaning to it and succeeding in connecting it to other areas in life represents an important 3^rd^ stage towards UE passion development and long-term UE engagement.

### Theme 4: UE as a lifestyle choice

UE training is a very time-consuming hobby. It requires a lot of discipline and very good time and energy management. It also means making choices in the allocation of available time according to one´s priorities. Making these choices explicit can create tensions, especially when own preferences conflict with the norm and social roles.

#### UE habit

All participants acknowledged the difficulty of maintaining balance between sport, work and family/private life as one of the main challenges. The resolution of this challenge was found in creating routines and habits around training leading to structuring the day, week and year according to training and racing schedules. This means that UE training is fully integrated into the daily routine, it becomes a habit and ultimately a lifestyle choice, which transcends all other choices in life. Even travelling becomes “Ironman tourism”, as one of the participants described it.

*“I would wake up super early on Sunday*, *drive to the mountains and try to be back for lunch*. *It has been like this for last couple of years*.*”*

*(A5)*

*“If you really love doing this and if you love to continue doing this*, *it becomes part of your life*, *becomes ingrained in your lifestyle*, *so you don’t have to make a choice*.*”*

*(A3)*

*“I wanted to work less*. *I asked specifically*. *Because it was possible to work 80% and I thought why not do it if you can improve quality of life and also can follow your passions next to it*? *For me that sounds like a good trade-off–I have a little bit less money but more quality time*.*”*

*(A15)*

UE lifestyle means making sacrifices. Most have to cut other hobbies, make social events which are not related to UE hobby very limited or practically absent. For many, due to the volume of training required to prepare for UE races, practically all free time is dedicated to UE training. Some embrace it consciously and fully, some struggle.

*“I was always gone for 3-4hr bike rides*, *coming home completely destroyed*, *and then you don’t feel like cleaning the house after*. *It was sometimes also putting pressure on our relationship*. *My husband sometimes said*: *you don’t have time for me*, *always with your sport*, *you are always gone*, *you go to work*, *then you go to sport and we never see each other*.*”*

*(A15)*

*“Now this became most part of my day on my weekend*. *It took a lot*. *I gave up other hobbies*. *Social life is very limited now*. *If I go out*, *I am the first one to go home because I am the 1*^*st*^ *one to get up*. *I feel sleepy and tired*. *But no regrets”*.

*(A2)*

UE lifestyle also includes adaptation of living conditions to fit the UE hobby, with a preference for living outside of the cities. One athlete in our sample moved from London to the Pyrenees in France, leaving his corporate job to become a full time UE coach and seeking better conditions for his training. He said:

*“My parents thought that leaving my corporate job and doing coaching full time*, *is maybe not the best financial decision I have ever made*. *But it was not a financial decision*, *it was a lifestyle choice*.*”*

*(A11)*

#### Mastery competence

All interviewed athletes were motivated by improving their skills to better handle the complexity and extreme difficulty of UE races. Many were engaged in deliberate practice: they followed an individual training plan, focused on and planned for the A-race of the season, or even a longer-term goal, and some worked with a coach.

*“I use a spreadsheet on Excel where I enter all my key sessions*, *usually 1 session per sport per week*, *and then I adapt the rest*. *I plan the session–I start with the race and then I go back*. *For example*, *I want to hit 4-5x 40min at this watt 2–3 weeks before Ironman and then I plan it back*. *Every block of training*, *I put main session every week but not always with specific power and pace target*. *And every Sunday night I plan my week*. *And I use HRV to get the idea for the recovery*. *I think I would probably be better with a coach*. *But I don’t want to let go of the freedom and learning about training*. *And it is also very rewarding to have a good race and qualify and to know you did it all by yourself*.

*(A6)*

All interviewed athletes had been progressively increasing the UE challenge year on year by racing longer and harder races. There was also a broad agreement that achieving mastery takes years and that with master competence a successful performance becomes more likely. All participants showed high motivation levels to keep going on their UE journey in the pursuit of mastery. This creates a powerful loop of positive reinforcement: progression towards mastery is satisfying and confidence-building. And the UE hobby becomes a passion.

*“When you have not done it yet it seems completely insane*. *Once you´ve done it*, *you think*, *well*, *it is only a 100km*.*”*

*(A4)*

*“I am more proud about the progression*, *step by step*… *it gives me confidence*.*”*

*(A7)*

*“I feel sorry for the people who don’t have passion*. *When you are driven by passion life has a different quality*. *It is very passion based*.*”*

*(A1)*

Establishing the UE hobby as a lifestyle choice and habitualizing it and the pursuit of UE mastery as a life-long commitment signify another important stage on the path of long-term UE passion development.

### Theme 5: UE passion and outcomes

#### Developing UE identity

With progressing mastery and developing UE lifestyle, athletes seem to define themselves more and more via UE. This does not mean that they forget their other social roles as parents, partners and professionals, but UE plays an important role in their self-definition. In this process of adult identity formation, UE acts as a psychological resource, an antidote and a compass for navigation in the post-modernist and post-industrial world, thus fulfilling some of the meta-needs.

*“I have never felt that I gave anything up for that*. *For me*, *it has become a part of my identity*.*”*

*(A7)*

*“I want to be able to do things that other people can’t do… I want to be able to run to a mountain bigger than somebody else*. *And it is part of my identity*. *If I look in the mirror and I ask myself*, *what do I stand for*, *I stand for the guy that can do things that other people can’t do*.*”*

*(A7)*

In addition to the positive spiritual benefits of experiences in nature, acquiring a wholesome sense of identity and self-concept, creating a coherent life story and fulfilling meta-needs, there are also a number of ambiguous outcomes of UE passion.

#### Obsessive passion

Athletes reported on their mood being dependent on training and fluctuating motivation. Positive mood is connected to good training sessions and high goal motivation, whereas negative mood states are connected to periods of demotivation after accomplishing a goal or just being fatigued by training.

*“My wife loves me getting away for training and coming back*. *I am so happy when I get back*. *If I don’t go*, *I will be miserable around the house*.*”*

*(A7)*

*“When you stop training for some reason*, *like when you are sick or injured*, *then quickly you start to miss it*, *you get depressed easily*, *you get itchy*, *irritated with people more quickly*, *you get stressed more quickly*, *you drink more alcohol*.*”*

*(A10)*

*“I don’t think it is healthy*. *Why would it be healthy to run more than 100k*? *And it is also not healthy to do nothing*. *I would go crazy if I did nothing*. *It is unhealthy because of injuries*. *I had plantar fasciitis*, *knee pains and now Haglund´s syndrome*. *I find it really difficult with injuries*. *Doing nothing and don’t see a change*. *And then you start again and it gets better*.*”*

*(A13)*

Participants also talked about internal pressures to keep training and about self-esteem contingencies connected to racing. These internal pressures can take control and compromise autonomy of action, thus making the experience less fulfilling. This, combined with mood dependency, can be a sign of obsessive passion, when activity is pursued uncontrollably, in spite of negative contingencies, and the autonomy of action is lost:

*“All my life I have been forced to train*… *Well*, *I did not directly force myself but I always had to train*, *keep thinking about it*, *it is pressure*. *And sometimes you want to do something else and you can´t*. *And your life revolves around your sport*. *And I want it the other way around now–I want to decide when I want to do sport*.*”*

*(A8)*

#### Vanity

Engaging in UE sport accentuates body awareness. Being an outlier for sporting achievements builds an expectation (both internally and externally) of an athletic “super-body”. Being on a start line of Ironman can be an intimidating experience if someone does not possess this type of lean and muscular physique. Doing fit things is inseparable from looking fit, this is the idea that most participant seem to share, and they also admit their vanity and the importance of looking fit on top of being fit. This is what sets them apart from “average and normal” people and gives them a sense of belonging to the UE tribe.

*“I like to be fit*. *Look fit at least*.*”*

*(A8)*

*“I suppose*, *I am a bit vane*, *in the sense that you want to look fit*, *look well*, *look healthy*, *there is a bit of that that goes with it*. *I don’t think of myself like a sedentary 60 years old and I don’t really feel like that*. *So*, *I did not spend a lot of time thinking about it I just realize that is not me*, *but another group of people*.*”*

*(A10)*

Preoccupation with own health and looks and the desire to “bio-hack myself” can intensify and put additional pressure when the first signs of aging start to manifest themselves and performance declines. Some embrace it with curiosity and openness, some with anxiety and despair.

*“Yes*, *I would like to hit triple digits*. *I want to be really strong through my 80s*. *I want to be fit*. *Many people just disregard the preparation you need to do to go through your 50s and 60s to be healthy*. *That is really important to me*. *That is why I look into sleep and nutrition*. *I want to make sure that when I get to my retirement*, *let´s assume it is 60*, *these 30 or 40 years I want to still be doing stuff*. *That scares me*. *So*, *if I don’t deal with it now*, *I don’t want to start thinking about it when I am 60*. *So that is important*.*”*

*(A7)*

*“Age started to be a huge factor*, *after 59 or so*. *Age is not progressive*, *it has huge jumps*, *I found it out the hard way*.*”*

*(A3)*

Obsessive passion can also become a path to unethical behaviour, when too much ego is invested into the passionate activity. One of the participants, a seasoned Ironman athlete, acknowledged the risk:

*“The issue of performance enhancement (doping)*, *it is a very thin line*. *And I think it is intrinsic to this sport*. *We spend so much money*, *so much ego*, *so much time on it*.*”*

*(A9)*

#### UE outlook to life

The UE lifestyle and adoption of UE habits signify a stage of full integration of UE into one´s identity and the achievement of the long-term sustainability of UE passion as a “life plan”. All interviewed athletes demonstrated not only strong commitment to UE, they actually talked about their life-long plans for an active endurance lifestyle. They envisioned themselves staying active for life, and, health permitting, still being super active and even racing in their 80s. While admitting, that the type of activity will most probably need to adapt to aging, they all had plans to swim, bike, run, ski, walk and move a lot … till they die. And some even made plans to die in a race.

*“My family knows that I need it*. *They have a clear idea that I will be in a race every year of my life for the rest of my life*.*”*

*(A14)*

*“I made this decision after Kona*, *it took me a couple of months*, *I want to be in the 70 to 74 age group*. *I want to make it to 74*.*”*

*(A3)*

*“I will always go to the mountains*. *Maybe not running*, *but climbing and hiking*. *I can’t imagine life without being out and in the mountains*.*”*

*(A12)*

*“I hope I will want to do sport all my life*. *I hope I can do it as long as possible*.*”*

*(A8)*

*“Hopefully I will die in an Ironman race*. *After 30km in marathon*.*”*

*(A1)*

### New temporal framework for progressive UE engagement and passion development

Of course, we hope that nobody will die in the UE race and that a harmonious passion of UE will enrich the lives of UE athletes for many years to come, to continue the positive and inspirational (life) stories we heard in the interviews. These stories, and commonalities between them, as well as the themes and subthemes generated in our analysis, led us to the creation of a holistic model for long-term UE engagement and passion development, Fig 2. We call it “A temporal framework for progressive UE engagement and passion development”

This framework connects the themes and milestones of the UE journey with the Self Development Theory of Behaviour, which was the meta-theoretical foundation for this research, and its key constructs of need satisfaction and motivations. It outlines the possible mechanisms and processes of UE passion development as well as its outcomes. As such, this model represents the psychological and phenomenological interpretation of the typical UE journey, based on the phenomenological and psychological interpretation of the qualitative data of this research and the most advanced research in UE psychology.

## Discussion

The growing popularity of extreme endurance and adventure races is a phenomenon of our post-industrial, post-modernist and very comfortable Western world. As a reflection of the society living in this world, it is complex from psychological, sociological and philosophical perspectives. With the current research we attempted to better understand the long-term UE journey and the passionate engagement development. We found that this journey is a long and rich one, full of peak experiences, emotional rollercoasters, overcoming and surrender stories and also of spiritual growth. It is a road less travelled—and it is a road of passion.

The IPA revealed 5 core themes and 13 sub-themes in the accounts of 16 individual UE journeys (Fig 1). These themes represent the unique and extra-ordinary lived experiences and their meanings in a temporal perspective from the moment of inception to a developed UE passion, practiced lifestyle and internalized UE identity. We further organized this journey in the temporal UE journey framework, presented above in Fig 2.

Whether someone is attracted by the idea of extreme racing or whether one attaches meaning of threat or opportunity to possible novel experiences, is probably defined by their high-level psychological predisposition [54, 55]. The reasons to engage in UE can be complex and this engagement cannot be predicted. It can be early childhood sport engagement, parental or social influence, natural inclination to extreme sports, an inborn or acquired set of character traits, or (and most likely) a combination of all of the above. We hope that further research will shed more light on the “making of” UE athletes. However, the findings of this research indicate clearly that a positive first UE experience plays a crucial role and represents the first milestone in the formation of a long-term passion.

“Tribe”, in anthropology, describes a notional form of human social organization based on a set of smaller groups, united by common descent, language, culture and ideology [56]. Social cohesion and sharing common values and norms are key features of a tribe and this is what individual UE athletes are looking for when they strive to resolve the social paradox inherent to their extreme hobby: their lifestyles and choices of pastime do not comply with those of their peers, and they feel frequently ridiculed and even marginalized. The most common cliché is that of being “crazy”. An answer to this is the formation of the UE sub-culture, which features different norms, where running 100km, cycling 200km and swimming 5km is expected, where training camps are considered to be holidays and where the “your training is my warm-up” mentality is widespread. In groups of like-minded people, social norms get re-framed and deconstructed and so the perceived “abnormality” of the UE hobby gets normalized and athletic identity gets activated and reinforced. We believe that embracing the UE sub-culture and finding “my tribe” and thus resolving the social paradox inherent to the UE hobby, represent a second major step on the UE journey.

Whether UE sub-culture is entering popular culture through the growing popularity of UE is however questionable. We think that it is rather striving to escape its normalization, which to some extent happened to marathon running. It does so by becoming even more ultra and, in fact, über-ultra, by adding longer and harder races around the world to get lost in the wilderness and quench the thirst for “more than ordinary moments of existence” [57] and peak experiences.

Peak experiences are "rare, exciting, oceanic, deeply moving, exhilarating, elevating experiences that generate an advanced form of perceiving reality, and are even mystic and magical in their effect upon the experimenter.” [57]. They are central to understanding the psychology of UE athletes and the stories about peak racing experiences represented the core of the conducted interviews. Their analysis produced 4 sub-themes: intense emotions, bodily experiences, self-discovery journey and uniting with nature. Polarity represents a joint feature of all sub-themes, since UE racing is an endless rollercoaster of positive-negative emotions and embodied experiences. The changeability of vitality states [18], pain tolerance [19, 20] and mental toughness [12, 13] of UE competitors have been well studied. Many studies highlight the complex and seemingly contradictory balance between extreme physical exertion and feelings of elevated mood and comfort [58]. This dichotomy produces optimal performance, or flow, which is a high challenge-high skills situation with the person being fully immersed in it and enjoying the process, stretching his/her skills and learning new ones, increasing self-esteem and personal complexity [59]. The newer research on optimal performance, however, makes a distinction between flow and clutch states [60], with the latter being closer to the UE racing and training experiences. A clutch state is described as a more effortful state than flow, with increased intensity and awareness of the situation and with more fixed than open goals, for example “just to finish/get to the top of the mountain” vs. “do my best/continue and see what happens”. Based on our observations, we would rather speak of a chain of adaptable fixed and open goals, which get fine-tuned in the progress of a long UE race (or training) as fatigue and reality set in. Thus, the athletes could move from a goal of finishing the race to “just getting to the next aid station” as they go through a variety of vitality states, flow, clutch and non-flow states, as well as some “dark moments”–a kaleidoscope of intense emotions and bodily experiences present in the dynamic phenomenon of UE training and racing, which requires a highly differentiated and also empathic study.

A less known aspect of UE experience is the “self-discovery journey” uncovered in our research. For participants, it was a very important long-term process, which was triggered by the “life story” format of the interview and the use of the timeline as a tool stimulating self-reflection. All participants alluded to the benefits of UE for personal development, of how the extreme experiences in races helped them to build self-confidence, learn humility and gratitude and even to become better people overall. While “self-discovery” can be seen as a cognitive process with pragmatic benefits, the “uniting with nature” sub-theme provides a spiritual dimension to UE racing. Accounts of mystical visions, unseen natural beauty, realizations about the sacredness of life and its purpose and meaning, were among the most moving experiences in the lives of interviewed athletes. Indeed, research confirms that UE athletes have higher scores in life meaning than runners competing in shorter race distances [61]. Getting to the top of a mountain after 100 or more km of tough racing, alone, in a sunset or sunrise, seeing an alpine ibex greeting you there, is likely to induce a sense of elation, magic and divine, especially when intensified by bodily experiences, physical exertion and central neural fatigue.

According to Maslow´s investigations into the peak experience phenomenon, particular settings and activities act as potent triggers for peak experiences and flow states, and they include solitude, physical accomplishment, meditation and being in nature, in particular water, sunset, mountains and wild animals [57, 59]. That wilderness can be a subject of scientific enquiry was proven by the popularity of the psychology of wilderness research in the 70s, 80s and 90s of the last century [62–65|. However, wilderness experience in the context of UE phenomenon is a novel angle and an interesting field for more research. Several interviewed athletes mentioned that being (deep and frequently alone) in nature is one of the key reasons of their engagement in UE sports.

Peak experiences make life worth living. They turn hobby into a passion. For all interviewed athletes, UE training and racing represented an activity about which they were passionate–and which satisfied their innate psychological needs of autonomy, relatedness and competence [32]. The autonomy need is fulfilled every time an athlete trains and races as an act of free volition–which is 100% of the time for all non-professional athlete (unless there are contingencies due to the obsessive type of passion). Relatedness is fulfilled by being a part of an UE tribe and by participating in races, which, contrary to popular opinion, are highly social events. And the decades or even life-long training and pursuit of UE mastery fully deliver on the competence need. When mastery skills are practiced in a self-directed and autonomous manner, they lead to flow states and peak experiences. Mastery represents a very powerful driver coming from within and a type of intrinsic regulation defining the self-identity. With mastery competence developed, a successful performance becomes more likely, which in its turn enhances intrinsic motivation, whereby autonomy and competence needs are met [66]. This all creates a powerful loop of positive reinforcement: UE becomes a high value need leading to repeated behaviour and increased engagement. The UE hobby becomes a passion. And the passion becomes a lifestyle. This lifestyle is based on UE identity, habit and master competency. Through the (unconscious) need satisfaction in UE hobby practice, athletes start internalizing it in their self-identity and start thinking about themselves as a “trail runner”, “athlete” or “triathlete”. The UE hobby becomes a self-defining activity, which assists them in exploring their sense of meaningful and coherent identity and providing a unique context for exploring their interest and talents [67]. Forming an adult identity in the post-industrial society is a challenge. It requires organizing one’s many experiences, roles, and personal values into a unified and purposeful whole [68]. The pursuit of a passionate activity, the interpretation of experiences stemming from it according to one´s disposition and the construction of one´s life according to it, allow UE athletes to create a unified and consistent adult identity, where UE takes a central role. “Living UE” becomes a habit and practicing it for many years develops a master competency.

Living UE passion and practicing UE lifestyle brings about a number of outcomes, which quality is driven by the type of passion formed. A harmonious passion is formed if the internalisation process is fully autonomous and internally motivated. If, however, this internalization is controlled and subject to external pressures, and too much ego is invested into the passion, an obsessive passion will be the result. An obsessive passion is connected to possible negative outcomes [31]. The participants talked about mood dependency on exercise, sacrifices in other hobbies and social life, and training through injuries among negative consequences of their passionate engagement and identity investment into UE. They also acknowledged vanity and exaggerated bio-centrism as characteristics of UE sub-culture, as well as the ego-centrism and perfectionism of many athletes. A harmonious UE passion brings about positive outcomes, such as a healthy lifestyle, balanced training, meta-needs fulfilment and the formation of an inspiring life-story.

Even more, peak experiences, need satisfaction, the UE tribe, identity and lifestyle, master competence in UE training and racing, all this leads to the formation of a passion-driven life plan for non-professional UE athletes: they simply do not see a reason to stop. Ever. They all claim to want to continue training, racing and leading an active lifestyle till a very old age, until they die, for some–preferably in a race.

### Limitations

Within the time constraint of maximum 2 hours, we were looking at many years of UE engagement with our participants. While this approach provided spontaneous “unaided” responses, some preparation time and advance notice for the participants might have produced more detailed timelines, which was also suggested in our member reflections, and resulted in more co-creation, true to the spirit of constructivist inquiry. Additional follow-up research with the same group could provide further insights for the “Temporal framework for progressive UE engagement and passion development” we created. In general, a longitudinal approach to qualitative and quantitative studies of the UE phenomenon would greatly enrich its understanding.

## Conclusion and perspectives

Like Tolstoy, who stated that “all happy families are alike”, we also found that there were similarities in stories of 16 UE interviewed athletes. Starting with an innate predisposition and intrinsic motivation towards endurance sports and adventure experiences (which can manifest itself early or later in life), UE athletes go through years of training and racing developing their passionate engagement. Over time, they satisfy more and more of their innate psychological needs through it, so that UE becomes a high value need leading to repeated race participation. By going through extreme physical and emotional experiences, athletes deepen their intrinsic motivation and solidify formation of the UE passion. This passion gets externally reinforced by the UE sub-culture. In parallel, the athletic UE self-identity is activated, developed and confirmed, so that the passionate hobby becomes an identity-defining activity and a lifestyle choice with a number of adaptive and maladaptive outcomes, associated with harmonious and obsessive passion types respectively.

And while the races and details of engagement with UE sports of our athletes were different, as much as their age, gender, social and cultural backgrounds, we found that the key high level milestones of their UE could be generalized. Thus, we stepped out of the IPA method and undertook an attempt to create a model of a long-term UE engagement and passion development. With this model we endeavour to create a comprehensive framework to understand the whole UE athlete, for which we combined our research results with the most advanced research in UE athlete psychology. We call it “Temporal framework for progressive UE engagement and passion development” and would like to invite the academic community to discuss it. We also invite sport psychologists and therapists to take a wholistic view on non-professional and passionate UE athletes and provide guidance and interventions considering the context of the whole journey and their identities. Further, adding an “outside perspective” would be most valuable, starting with the entourage (family and friends) of the athletes.

We believe that UE helps athletes to navigate their lives in our post-industrial and post-modernist world in the pursuit of a coherent self-identity, satisfaction of their meta-needs, happiness and fulfilment.

## Supporting information

S1 Fig Timeline of participant A1. (TIF)

S2 Fig Timeline of participant A2. (TIF)

S3 Fig Photo collages interview. (TIF)
