# Supplementary figures and images for: A new temporal framework for the passionate engagement journey of ultra-endurance athletes: A qualitative investigation
Source: PLoS One. 2023 Nov 27;18(11):e0293864. doi: 10.1371/journal.pone.0293864 (PMC10681185; doi:10.1371/journal.pone.0293864)

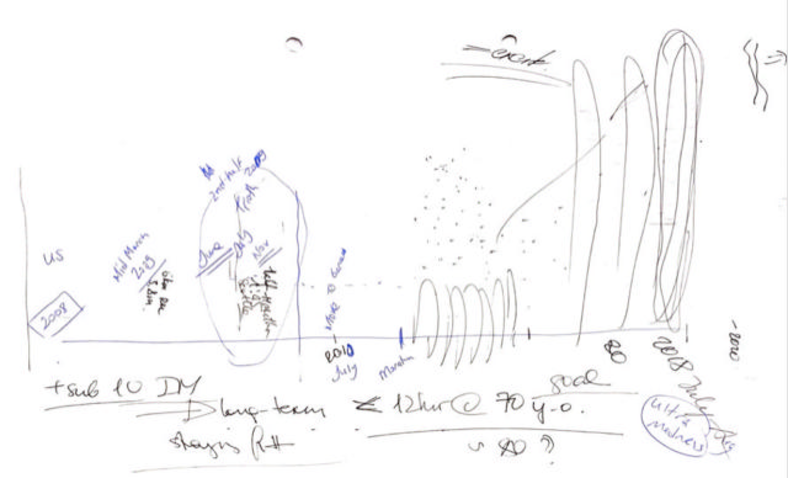

Supplement: S1 Fig — (TIF) [file pone.0293864.s001.tif]

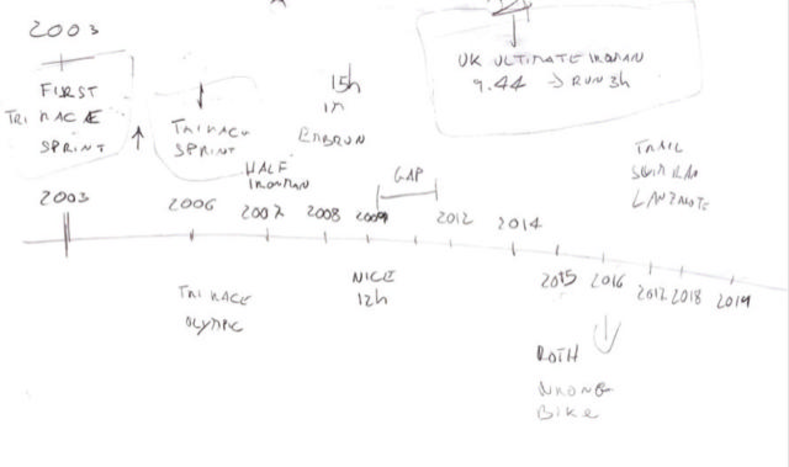

Supplement: S2 Fig — (TIF) [file pone.0293864.s002.tif]

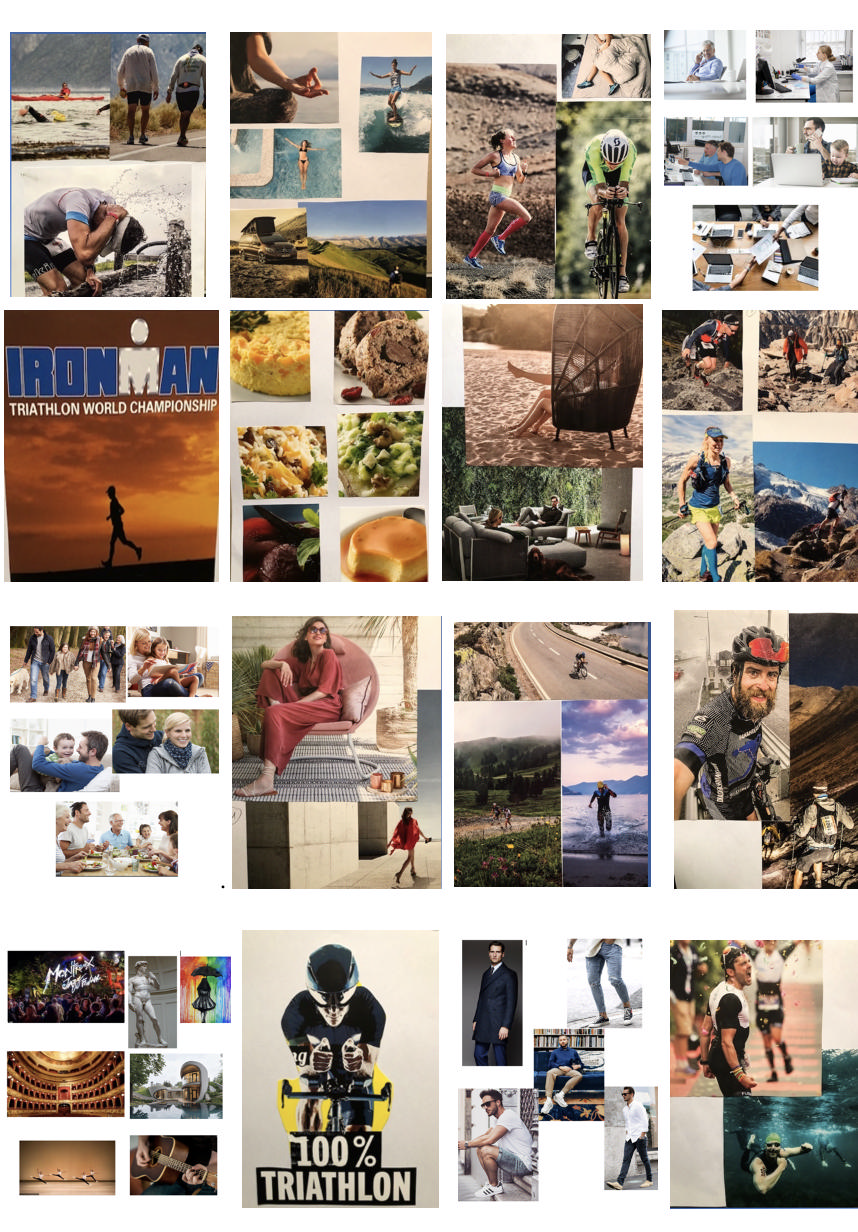

Supplement: S3 Fig — (TIF) [file pone.0293864.s003.tif]
